# A Comprehensive Neurorehabilitation Program Should be an Integral Part of a Comprehensive Stroke Center

**DOI:** 10.3389/fneur.2014.00057

**Published:** 2014-04-22

**Authors:** Reza Bagherpour, Dennis D. Dykstra, A. M. Barrett, Andreas R. Luft, Afshin A. Divani

**Affiliations:** ^1^Department of Physical Medicine and Rehabilitation, University of Minnesota, Minneapolis, MN, USA; ^2^Department of Neurology, University of Minnesota, Minneapolis, MN, USA; ^3^Stroke Rehabilitation Research, Kessler Foundation, West Orange, NJ, USA; ^4^Clinical Neurorehabilitation, Department of Neurology, University of Zurich, Zurich, Switzerland; ^5^Department of Neurosurgery, University of Minnesota, Minneapolis, MN, USA

**Keywords:** neurorehabilitation, stroke, comprehensive stroke center, neurological recovery, depression

## Introduction

With the aging population, strokes have become a leading cause of disability and cognitive impairment. On average, someone has a stroke every 40 s in the United States, resulting in approximately 800,000 strokes annually (1, 2). As a result, acute management of stroke volume has increased in order to meet the demand. This includes initial assessment, diagnostic imaging, laboratory studies, and appropriate acute treatments (3).

Forty percent of stroke survivors experience moderate to severe impairments requiring specialized care (4), while about 10% will require long-term care or placement in a skilled nursing facility (5). Furthermore, even minor strokes are associated with memory, spatial, and mood disorders as well as other hidden disabilities (6). Morbidity from stroke remains high despite new advances in treatment of acute stroke with thrombolytic agents and endovascular approaches (1). Therefore, protocols for appropriate triage and referral at all care stages may need to be centralized. An ideal setting for implementing systematic care protocols is a comprehensive stroke center (CSC).

## Integrating Neurorehabilitation Service in CSCs

Although CSCs treat acute stroke patients more effectively (7), we feel that a critical part of the management process is often overlooked by the lack of emphasis on neurorehabilitation. As a result, delay in initiation of pathways for evidence-based and targeted rehabilitation care can occur, even in these specialized settings.

The ideal way to integrate neurorehabilitation services in CSCs is to base recommendations on quality improvement studies that have demonstrated how to manage referral protocols to optimize rehabilitation outcomes. For example, studies examining the impact of referring patients for rehabilitation during the first days of admission vs. those referred at discharge can be used to create quality standards for internal and external monitoring. Unfortunately, studies comparing rehabilitation outcomes with different rehabilitation care referral procedures are not yet available. CSCs are ideal settings for examining differences in outcome based on systems of care, and thus we urge stroke researchers to begin evaluating and comparing rehabilitation referral pathways. However, until evidence-based protocols for rehabilitation are available, true quality monitoring in the CSC setting needs to be based on the best-practice standards.

Psychological care is an integral part of all neurorehabilitation programs, due to the fact that stroke patients are at high risk of depression (8, 9). Integrating rehabilitation into a CSC will facilitate psychological and psychiatric evaluation of all stroke patients (10).

## Benefits of Availability of Neurorehabilitation Service in CSCs

The presence of neurorehabilitation services in designated CSCs can allow for the continuity of care from physiatrists, neurologists, rehabilitation nurses, physical and occupational therapists, speech-language pathologists, dieticians, social workers, neuropsychologists, case managers, and recreational therapists as part of the experienced and specialized interdisciplinary team paying careful attention to neurorehabilitation. With a more systematically integrated assessment of progress, including attention to psychosocial issues and early comprehensive discharge planning, this model for stroke-care not only potentially improves patient outcomes, but also decreases the financial burden on the medical care system and improves hospital–home transitions.

Rehabilitation should begin in the hospital, as soon as possible, following the stroke. Any rehabilitation program should aim to improve function by allowing stroke survivors to operate as independently as possible (11). Stroke sequelae invariably include both neurological impairments and related functional disabilities (12–14). Early spontaneous neurological recovery is dependent on local processes leading to initial clinical improvement independent of behavior or stimuli. Functional recovery is influenced by both rehabilitation interventions and spontaneous neurological recovery. Therefore, an effective neurorehabilitation regimen can be extremely beneficial to both types of recovery.

Since peak neurological recovery occurs within the first 3 months of the initial insult (15) and large numbers of stroke survivors may not be able to access outpatient treatment (16), it is essential to expeditiously incorporate a comprehensive neurorehabilitation regimen as part of any universal stroke treatment curriculum. Neurological reorganization plays an important role in this restoration of function. It can extend for a much longer period than local processes, such as the resolution of edema or reperfusion of the penumbra. Of particular interest is the influence of rehabilitation training on neurological reorganization. For example, motor imagery neurorehabilitation techniques that have long been used for athletic improvement (17) are a feasible treatment for patients with sensory-motor impairments following a stroke, and may also support sensory-motor reorganization to prepare for the return of function (18). Techniques such as these might be used early in the recovery period while reorganization is concurrently taking place (19).

Another major advantage of involving rehabilitation in CSCs is to initiate a robust rehabilitation care pathway that includes post-acute, home-based and chronic components, patient and family education, and lifestyle adjustments (see Table 1). A large number of stroke survivors can benefit from inpatient acute rehabilitation in hospitals that provide a full range of rehabilitation services combined with skilled nursing staff. These are generally the only settings where subspecialty rehabilitation providers and intensive treatments are available.

**Table 1:** **Brief outline of care pathway for neurorehabilitation after stroke, Adapted from Ref. (20, 21)**.

Care stage	Hyper-acute	Acute	Post-acute	Home-based	Outpatient
**Time after event**	Minutes to hours	Days	1–3 months	Varies, but may be 3–6 months	6 months and beyond
**Includes options (but not limited to)**	Recombinant tissue plasminogen activator, surgical/mechanical approaches	Pharmaceuticals to modify thrombotic and embolic stroke risk, reduce risk of complications and promote neural recovery	Behavioral and pharmaceutical treatments to promote neural reorganization, and treat depression	Nursing care to optimize the home and survivor–caregiver interaction, exercise therapy	Behavioral and pharmaceutical treatments to promote neural reorganization, exercise therapy, learning compensatory strategies
**Recovery stage**	Neural recovery	Neural reorganization	Neural reorganization	Neural reorganization	Neural reorganization, also use of compensatory strategies
**Rehabilitation management**	Begin clinical assessment of new limitations	Complete clinical assessment, identify short- and long-term rehab goals, introduce neurorehabilitation care pathway stages to patient and family and begin arranging care transitions, initiate first part of treatment plan	Develop short-term goals, continue treatment plan, re-assess clinical status, monitor progress and revise long-term goals, interpret treatment plan to patient and family	Same as post-acute, but also implement community re-integration goals, identify and engage community-based support	Same as home-based, continue engagement of community-based resources (e.g., vocational, driving training)

Long-term care facilities, home therapy through visiting nursing staff, day programs, and outpatient therapy are all viable options for the next stage of recovery, which may continue for years, albeit at a less rapid rate. As opposed to daily rehabilitation offered in acute inpatient facilities, other facilities offer rehabilitation services two to three times per week to optimize activity of daily living to prevent decline in functional ability and performance. As part of the transition to the community, access to appropriate support services (vocational counseling, peer advocacy, and social support organizations) is extremely important.

Because current stroke-care frequently involves a delay in initiating intensive acute inpatient rehabilitation, starting these services within a CSC, to continue in another sub-acute setting, may be optimal for qualifying patients (22). As these acute services may favorably modify motor, communication, or other recovery trajectory, we can expect that (faster) patient recovery to baseline functional status will result in significant reduction in healthcare costs.

## Standardized Measurement for Disability and Outcomes for Rehabilitation

The importance of evaluating disability outcome measures is well recognized in patients undergoing inpatient neurorehabilitation (23). Prior to initiating a rehabilitation regime for stroke patients, it is imperative to incorporate a uniform system of measurement for disability based on the International Classification of Impairment, Disabilities, and Handicaps (24). Furthermore, the use of a standardized classification system to categorize the level of deficit, disability measure, or resulting long-term handicap allows for a better selection of the patient population for clinical trials (25).

We propose that all stroke patients be initially evaluated by the functional independence measure (FIM), or its short version (AlphaFIM) that is created for acute care settings (26), just before discharge from a CSC, as well as at the start and conclusion of rehabilitation therapy (which is currently the care standard). Acute evaluation can help clinicians design treatment programs more precisely to predict outcomes of rehabilitation treatments.

Although many acute stroke survivors show some level of improvement regardless of treatment, we urge our colleagues to plan quality improvement and care feasibility studies to determine how co-treatment with rehabilitation can be most effectively managed in CSC settings.

## Development of Neurorehabilitation Guidelines for CSCs

Since the inception of physician quality research, the Center for Medicare and Medicaid Services has evaluated referral to rehabilitation as a quality care measure in stroke and stroke rehabilitation, however, no requirements have been specified about the process of rehabilitation evaluation. It is inevitable that these specifics should be defined and appropriate care processes be implemented as part of best-practice stroke-care. At this time, we feel that involving highly qualified subspecialists in rehabilitation in CSC rehabilitation referrals is the best way to enforce a clinical practice standard. The United Council for Neurologic Subspecialties (UCNS) certifies neurologists and physiatrists in neurorehabilitation. Starting in 2014, the American Board of Physical Medicine and Rehabilitation (ABPMR) will certify neurologists, physiatrists, and family practice physicians in Brain Injury Medicine. These specialists are the most appropriate experts to manage rehabilitation referrals. We also feel they can assemble the guidelines for an effective post-stroke neurorehabilitation regime to be implemented across stroke-care settings. The potential increase in efficiency and improvement in access to a multifaceted care regimen in turn justifies the need for more CSCs that offer neurorehabilitation services.

Some stroke survivors with severe disabilities, premorbid (or new) dementia, major illness, or unstable medical problems can have difficulty tolerating intensive exercise therapy. For these patients, modified inpatient rehabilitation care pathways can be used so as to focus on other areas of function (e.g., spatial-motor function, swallowing, truncal stability) and, after acute care or inpatient rehabilitation, care can be continued in sub-acute facilities that provide daily nursing care in association with other services (e.g., pain management).

Emerging treatments to support motor recovery include serotonergic antidepressants – in the FLAME study, empirical (prophylactic) treatment with fluoxetine may even promote recovery as compared to placebo (27). A recent Cochrane review including all selective serotonin reuptake inhibitors (28) also supported a beneficial effect of these agents on stroke recovery. This suggests the beneficial potential of treating depression extends to increasing mobility. Patients can also benefit from treatments for emotional disturbances and anxiety, which are routinely assessed during rehabilitative care. The shortcomings for current screening batteries for post-stroke depression should be noted as the assessment can be complicated by stroke-related cognitive and somatic deficits (29, 30). Therefore, there is a need for custom-tailored screening tools with higher sensitivity and specificity in assessing depression among stroke survivors.

## Cost-Benefit Consideration of Neurorehabilitation Service in CSCs

Published data pertaining to the cost-benefit analysis between neurorehabilitation services in CSCs vs. elsewhere are scarce. Most of the published studies are related to comparison between traditional inpatient rehabilitation and early supported (in-home rehabilitation) discharge (31–33). The outcomes of a few studies conducted in Europe have shown a lower cost, though not statistically significant, for the stroke patients who received their rehabilitation therapy in the stroke unit vs. those who received it in other hospital wards (34, 35). Future studies should not only look at the treatment cost, but also long-term cost related to improvement to quality of life including dependency and care costs.

Early mobilization and recovery acceleration are likely to reduce events such as falls, and reduce the incidence of hospitalization-associated delirium (36). Therefore, from an economic perspective emphasizing neurorehabilitation as an integral aspect of CSC treatment can potentially reduce burden of stroke-care. Moreover, integrated neurorehabilitation services may accelerate hospital discharge with a coordinated transition to home-based rehabilitation for selected stroke patients that can significantly reduce the cost of care without worsening the outcome (37). It is also important to mention that level I specialized neurorehabilitation services may incur higher cost mainly due to a high-level trained therapy staffing that is required to deal with a more complex caseload (38). Therefore, a proper triage by the CSCs medical staff can help to optimize utilization of such services.

## Summary

Comprehensive stroke centers should be the leading choice for treating stroke victims. Concomitant neurorehabilitation program should be an integral part of any CSC (39). A coordinated multidisciplinary rehabilitation within stroke units has been one of the components credited for long-term reductions in death, dependency, and need for institutional care (40). Integrating neurorehabilitation services and initiating rehabilitation care pathways with acute, sub-acute, home, and chronic components offers a CSC the opportunity to significantly improve patient outcomes. Via this structure, emerging treatment options such as constraint therapy for motor and language recovery, synergy of motor-language rehabilitation, and virtual feedback approaches, and non-invasive magnetic and electrical brain stimulation (41) can better customize therapy so that maximum recovery may take place. This allows for appropriate early rehabilitation, counseling of patients and families on sub-acute options, and takes action against preventable morbidity in the hospital and at the time of home transition. Health outcomes research in stroke needs to extend to studying rehabilitation interventions in order to evaluate optimal regimens for early intervention that are feasible in many settings, cost-effective, and well-accepted by patients and families.

## Conflict of Interest Statement

The authors declare that the research was conducted in the absence of any commercial or financial relationships that could be construed as a potential conflict of interest.
